# Foramen magnum meningioma: long-term follow up without neurosurgery—A case report

**DOI:** 10.1186/s13256-025-05725-x

**Published:** 2025-11-26

**Authors:** Marija Djukic, Johannes Gossner, Jörg Larsen, Veit Rohde, Roland Nau

**Affiliations:** 1 Department of Neuropathology, University Medical Center Göttingen, Robert-Koch-Straße 40, 37075 Göttingen, Germany; 2 Department of Radiology, University Medical Center, Göttingen, Germany; 3 Department of Neurosugery, University Medical Center, Göttingen, Germany; 4 Department of Geriatrics, Evangelisches Krankenhaus Göttingen-Weende, Göttingen, Germany; 5 Department of Radiology, Evangelisches Krankenhaus Göttingen-Weende, Göttingen, Germany

**Keywords:** Meningioma, Brain stem, Hydrocephalus, Magnetic resonance imaging, Computed tomography

## Abstract

**Background:**

Foramen magnum meningiomas can cause lower cranial nerve deficits or brain stem symptoms. When they become symptomatic or when growth is documented, surgical resection is indicated.

**Case presentation:**

In a white German 79-year-old woman, a small dorsal foramen magnum meningioma was detected by cerebral magnetic resonance imaging. After 7 years, cerebral magnetic resonance imaging showed considerable tumor growth (an increase in diameters from 15 × 16 × 20 mm to 28 × 30 × 37 mm), brain stem compression, and obstructive hydrocephalus. The clinical symptoms were however mild. The patient refused surgery. After a follow-up of over 18 months, she is still able to walk and live relatively independently.

**Conclusions:**

In the case of a slowly growing presumed benign intracranial tumor, deferral of surgery may be a justifiable option in old age, when symptoms are mild and social factors present an obstacle to immediate surgery. Since a spontaneous reduction in the growth rate is often observed in large intracranial meningiomas, some patients can survive with moderate deficits and a high quality of life in spite of substantial compression of the brain stem.

## Introduction

Meningiomas are frequent and most commonly benign intracranial tumors. Usually, they are slow-growing, and many meningiomas remain asymptomatic. Frequent signs and symptoms of meningioma growth are seizures and neurological deficits either related to compression of brain parenchyma, edema formation or, if located at the skull base, involvement of cranial nerves [1–3]. Rare complications leading to rapid deterioration are secondary intra- and peritumoral and other intracranial hemorrhages [4–6].

Foramen magnum meningiomas are rare, accounting for 1.8 to 3.2% of all meningiomas. As a consequence of their location, foramen magnum meningiomas can cause lower cranial nerve deficits or brain stem symptoms. When foramen magnum meningiomas become symptomatic or when growth is documented, surgical resection is indicated [2, 3]. However, the surgical morbidity is not negligible, especially, when the anterior foramen magnum is involved.

## Case presentation

### History

A 79-year-old retired white German woman with a past medical history of cholecystitis, gastritis, Herpes zoster, and wrist fracture presented with an unexplained sudden fall with a probable short loss of consciousness and a subsequent hematoma of her left thigh and hip. Upon admission, she was fully orientated and not depressive.

**Fig. 1:**
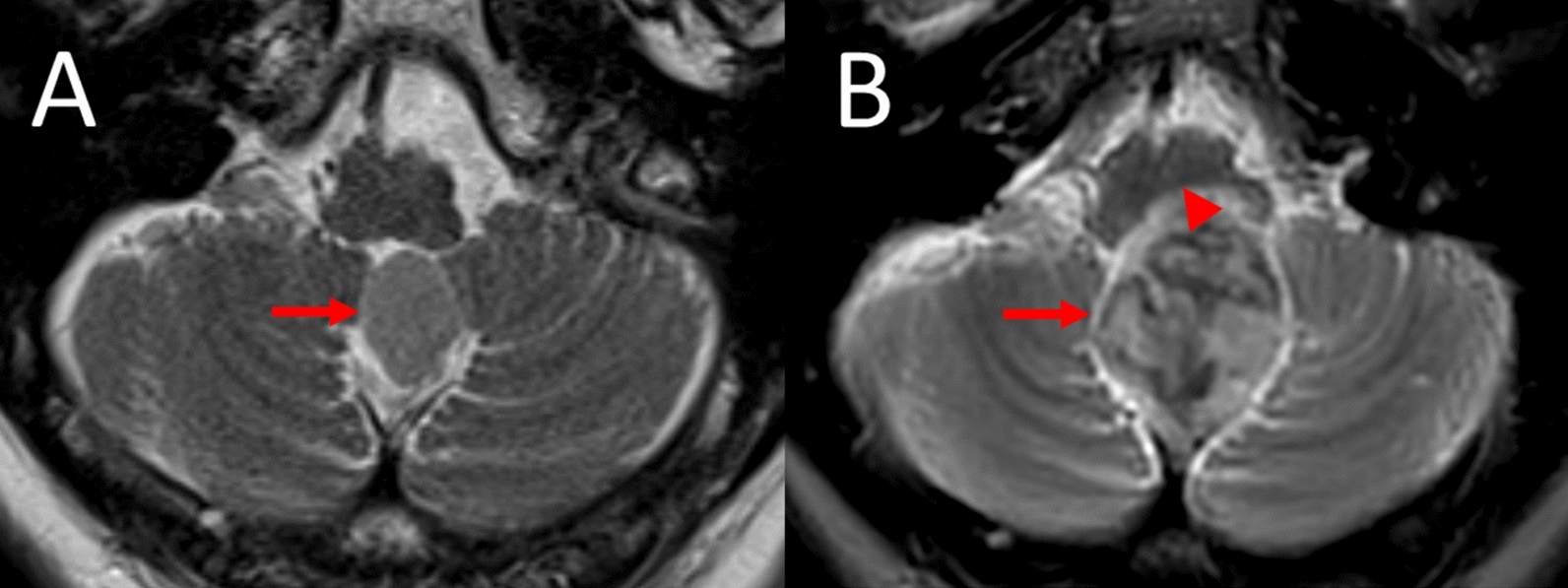
Growth of an infratentorial dorsal foramen magnum meningioma over 7 years (transverse-axial T2-weighted magnetic resonance imaging sections). **A**) Well-delineated subvermal midline tumor (diameters 15 × 16 × 20 mm) with homogeneous high-signal intensity on T2-weighting when compared with adjacent normal brain matter (arrow). **B** Tumor growth progression (diameters 28 × 30 × 37 mm) after 7 years (arrow); loss of tumoral homogeneity with new areas of signal loss. Please note new subpontine brain stem compression without cerebral T2-hyperintensity on the follow-up scan (arrowhead)

**Fig. 2:**
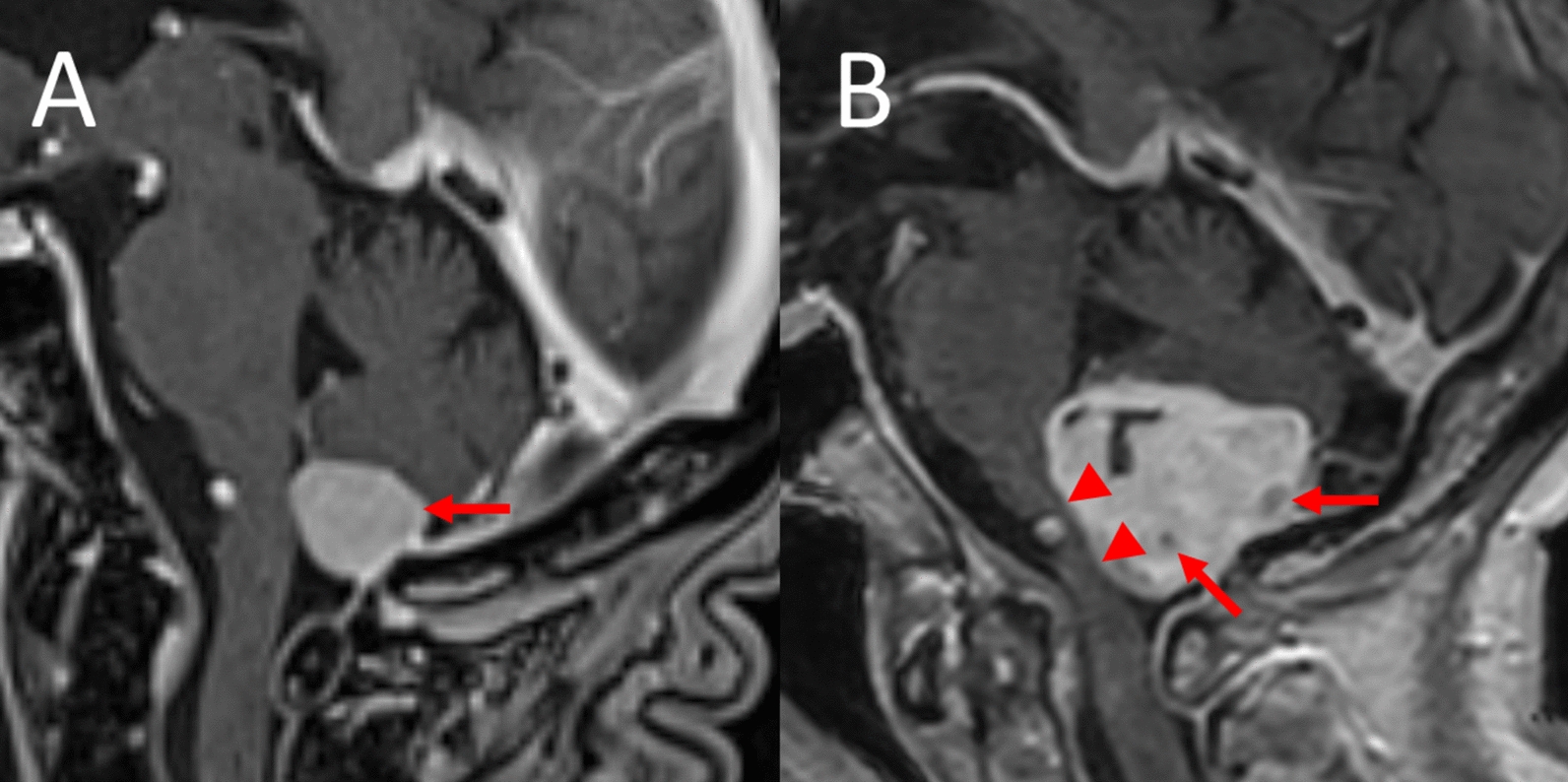
Growth of an infratentorial dorsal foramen magnum meningioma over 7 years (near median parasagittal T1-weighted contrast medium-enhanced magnetic resonance imaging sections). **A** Homogeneous avid tumoral contrast medium enhancement (arrow). **B** Tumor progression, inhomogeneous contrast medium enhancement with new areas of nonenhancement (arrows). Please note new subpontine brain stem compression on the follow-up scan (arrowheads)

The pretreating neurologist noted a palsy of the left sixth cranial nerve, which according to the patient’s opinion had been present from childhood, and an axonal distal-symmetric peripheral neuropathy of unknown etiology. On ultrasonography, no stenosis of the brain-supplying vessels was detected. Electroencephalography showed no epileptic activity. Magnetic resonance imaging (MRI) of the brain and the cervical spine showed a dorsal foramen magnum meningioma with a diameter of 15 × 16 × 20 mm (Figs. 1A and 2A) and two small meningiomas of the right sphenoidal wing and adjacent to the right precentral cortex, respectively. A moderate narrowing of the cervical spinal canal between the vertebrae 4 and 6 was also noted. The patient was advised to present for a follow-up examination 3 months later (Table 1).

**Table 1:** Timeline of foramen magnum meningioma

Clinical event	Patient’s age (years)	Intervention	Result
First symptoms	79	1 st cranial MRI	Dorsal foramen magnum meningioma, diameters 15 × 16 × 20 mm
Fall, hematoma of the left kidney	86	Transfusion of red blood cells, 2nd cranial MRI, physiotherapy	Considerable enlargement of the meningioma, diameters 28 × 30 × 37 mm
Follow-up at 2–3 months intervals for over 18 months	87	Physiotherapy, cranial CT 3 months after the 2nd MRI	Partial calcification, but no increase in the size of the meningioma, no progression of the mild hydrocephalus

*CT* computed tomography

*MRI* magnetic resonance imaging

For the next 7 years, the patient did however not attend a neurologist. After another fall and a hematoma of the left kidney requiring the transfusion of 3 × 500 mL concentrated red blood cells, she was admitted to our hospital.

### Clinical findings

#### Neurological exam

Upon admission, the patient temporarily showed a slowing of response speed. Her temporal orientation was not always exact. She was oriented to person, place, and situation. Except for the preexisting palsy of the left sixth cranial nerve, no further cranial nerve abnormality was noted. Nuchal rigidity was absent. Physical examination findings included a slight weakness of the elevators and abductors of the shoulder, the spreading of the fingers, the flexion of the hips on both sides, and of the extension of the left knee joint. The muscle tone was not elevated, the tendon reflexes including the Achilles tendon reflex were stronger on the right than on the left side. Babinski’s sign was absent on both sides. Hypoesthesia and hypoalgesia on the left arch of the foot and pallhypesthesia on both medial malleoli were noted. In Romberg’s test with open eyes, the patient fell backward after a few seconds. With closed eyes, she fell immediately. Limb ataxia was absent, and diadochokinesis was slowed on both sides. Gait was unstable, and the patient was unable to stand on one leg. She was however able to walk freely.

### Diagnostic assessment

#### Imaging

**Fig. 3:**
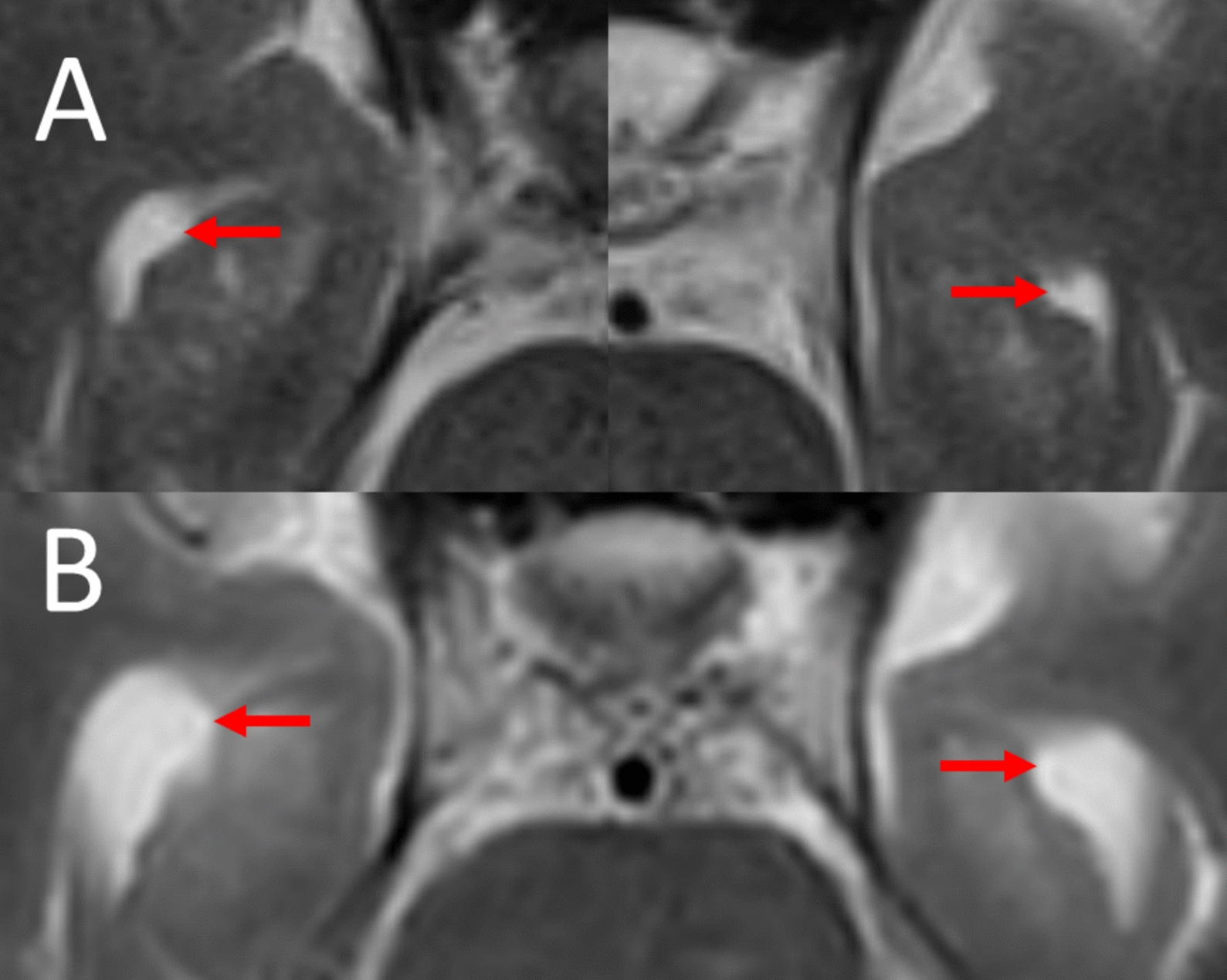
Hydrocephalus internus caused by an infratentorial dorsal foramen magnum meningioma after 7 years (transverse-axial T2-weighted magnetic resonance imaging sections). **A** Normal width of the temporal horns of the lateral ventricles (arrows). **B** Increase in the width of the temporal horns of the lateral ventricles in the follow-up scan indicative of obstructive hydrocephalus (arrows)

MRI now showed a considerable enlargement of the dorsal foramen magnum meningioma (diameters 28 × 30 × 37 mm) with compression and ventral dislocation of the brain stem, rostral dislocation of the cerebellum (Figs. 1B and 2B) and mild internal hydrocephalus. The radial width of the temporal horn of the lateral ventricles [7] had increased from 4/3 mm (right/left temporal horn) in the first MRI scan to 7/7 mm 7 years later (Fig. 3A and B). The size of the other two meningiomas of the right sphenoidal wing and adjacent to the right precentral cortex had not increased substantially.

#### Laboratory findings

As a consequence of the hematoma, upon admission plasma hemoglobin was 81 mg/L, and the patient received a transfusion of packed red blood cells. Thereafter, plasma hemoglobin slowly returned to normal. Plasma creatinine initially rose up to 21.1 mg/L, and then decreased to values around 11 mg/L corresponding to an approximate glomerular filtration rate of 45 mL/min. As a consequence of the resorption of the hematoma, plasma c-reactive protein and lactate dehydrogenase were temporarily elevated. No other severe laboratory abnormality was noted.

#### Neurosurgical consultation

Because of the compression of the brain stem and the unstable gait, in spite of the advanced age of the patient, the treating neurosurgeon recommended surgical removal of the foramen magnum meningioma. He estimated the risk of an aggravation of the neurological deficits subsequent to neurosurgery as less than 3%. As a consequence of the multimorbidity of the patient, the duration of the postoperative recovery was considered to be approximately 3 weeks. Because of the lack of brain edema, corticosteroid therapy was considered ineffective. Radiosurgery was not recommended because of the risk of swelling of the radiated tissue.

#### Decision making

After several in-depth consultations, the patient refused surgery because of her sick husband, whom she could not leave unattended at home during surgery and postsurgical rehabilitation. The couple has no children, and she felt that he might die in a nursing home during her reconvalescence time. She was therefore discharged home and received outpatient physiotherapy.

### Follow-up and outcomes

The patient has been seen regularly as an outpatient at 1–3 months intervals for 18 months:

Month 1: the patient complained of an unstable gait. She was, however, able to walk freely without aids. She reported the month correctly, but was unable to state the correct date and year.

Month 4: her gait instability had improved as a result of regular physiotherapy. In Romberg’s test, she was able to stand with open eyes for over 10 s, with closed eyes she fell after 9 s. She reported the month and year correctly, but missed the correct date by 1–2 days.

Month 6: she complained of upper back pain. She was fully oriented. In Romberg’s test, she was able to stand with open and closed eyes for over 10 s. Temporal orientation was intact.

Month 7: she wished to reduce the number of drugs prescribed. Acetylsalicylate, which the patient had taken for years without a clear indication, was discontinued. Her gait was stable with a 4-wheeled walker. Temporal orientation was intact.

Month 10: the patient complained of tiredness. She had stopped physiotherapy. In Romberg’s test, she was able to stand with open eyes for over 10 s, with closed eyes she fell after 6 s. She was able to walk freely. Temporal orientation was intact. Plasma creatinine had risen to 13.6 mg/L. The patient was advised to increase her fluid intake.

Month 11: the patient complained of neck pain. Temporal orientation was intact. Her 4-wheeled walker was incorrectly adjusted. After readjustment, she was able to walk safely. Physiotherapy, which the patient had not recommenced, was strongly recommended.

Month 13: the patient complained of lower back pain. Paravertebral muscle tenseness was noted. Conventional radiography showed no fracture of the pelvis or lumbar spine. In Romberg’s test, she was able to stand with open eyes for over 10 s, with closed eyes she fell after 7 s. She was able to walk freely. She missed the correct date by 2 days, but named month and year correctly. Physiotherapy, which the patient still had not restarted, was again strongly recommended.

Month 17: the patient complained of weakness and pain in the neck, shoulders, and upper arms. Erythrocyte sedimentation rate and c-reactive protein performed to exclude polymyalgia rheumatica were normal. In Romberg’s test, she was able to stand with open eyes for over 10 s. She was again able to walk freely. Plasma creatinine had fallen to 11.0 mg/L.

**Fig. 4:**
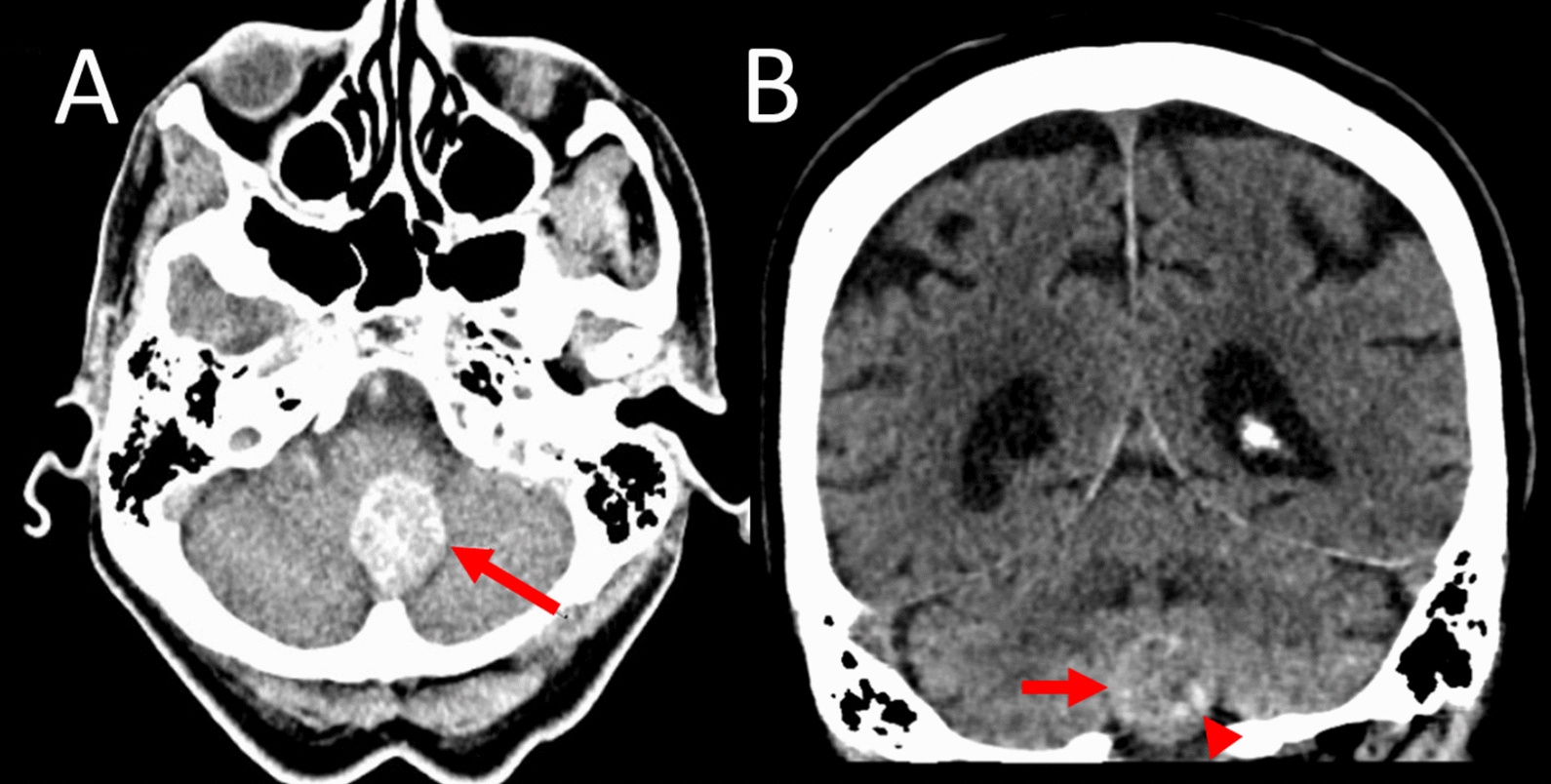
Infratentorial dorsal foramen magnum meningioma after 7 years and 3 months (cranial computed tomography). **A** Contrast medium-enhanced transverse-axial computed tomography section through the posterior fossa showing an avidly and homogeneously enhancing subvermal midline tumor (arrow) causing subpontine brain stem compression. **B** Transverse-axial computed tomography section without contrast enhancement showing the tumor (arrow) and inhomogeneous calcifications (arrowhead)

At all follow-up exams, the patient did not complain of headache, nausea, or vomiting, and had not developed additional focal neurological signs. A cranial computed tomography performed 3 months after the second MRI revealed partial calcification, but no increase in the size of the meningioma (Fig. 4B) and no progression of the mild hydrocephalus. The patient still lives together with her husband and continues to refuse surgery, because she does not want to leave her husband unattended.

## Discussion and conclusion

The present case illustrates that in spite of the considerable compression of the brain stem by a meningioma situated in the foramen magnum, some patients can survive without surgery with moderate deficits which do not prevent them from living relatively independently with a good quality of life.

In a retrospective study on 71 patients after surgery for intracranial meningioma and an age > 70 years, postoperative Karnofsky Performance Status Scale was lower, and 3-month (5.6% versus 0.3%; *p* = 0.003) and 1-year mortality (8.5% versus 0.3%; *p* < 0.0001) were higher in patients > 70 years than in younger patients [8]. Stereotactic radiosurgery is an alternative to surgery in meningiomas < 3 cm [2, 9–11]. In the present case, radiosurgery was not felt appropriate (a) since its effect on tumor size would not be immediate and (b) because of the size of the tumor. Following radiation, the tumor may initially swell, increasing its space occupying effect upon the brain stem. This swelling might have caused disastrous consequences.

Several studies have investigated the natural history of meningiomas. Most meningiomas grow [1, 12]. In a large retrospective study with a mean observation period of 46.9 ± 30.1 months, 159 of 240 (66.3%) of meningiomas demonstrated growth. A total of 75 meningiomas (31.3%) were stable in size, and 6 (2.5%) meningiomas showed spontaneous regression. In multivariable logistic regression, age at diagnosis (odds ratio [OR] 0.979 [95% CI 0.958–1.000], *p* = 0.048) was a mild, and calcifications in the tumor (OR 0.442 [95% CI 0.224–0.872], *p* = 0.019) a moderate predictor of absent tumor growth [12]. T2-weighted signal iso/hyperintensity and the absence of calcifications were moderate predictors of rapid growth in untreated intracranial meningiomas [13]. In our patient, age and the presence of calcifications enhance the chance of slow or absent further growth, whereas T2-weighted signal iso/hyperintensity is a predictor of further increase in tumor volume. Symptomatic and especially large and symptomatic meningiomas have been largely underrepresented in these series, because of the clear indication for surgery in these cases. In an autopsy study on 100 asymptomatic intracranial meningiomas found incidentally (9% in the posterior fossa) at the Mayo Clinic Rochester between 1939 and 1954, two large meningiomas (diameters up to 5 × 6 × 7.5 cm) were found [1]. One patient showed no progression of clinical symptoms over 14 years [14]. In approx. 2.5% of the cases, a spontaneous reduction in the growth rate of intracranial meningiomas has been observed [12]. Physicians in the last century—when perioperative mortality was higher than today—recommended, “if a patient presents with a fixed deficit that is static by history, and a presumed meningioma is identified during an appropriate workup, this patient should…be followed clinically and radiologically for signs of further functional compromise before neurosurgical intervention is contemplated” [15].

Our case illustrates that in spite of compression of the brain stem by a presumed meningioma, deferral of surgery is a justifiable option in old age, when the symptoms are mild and social factors present an obstacle to immediate surgery. The patient's wishes must guide the therapeutic approach, particularly when an intervention carries the risk of death or severe disability [16]. Therefore, after consideration of all options and necessities, a watch-and-wait strategy in the present conditions appears to be the adequate choice.

## Data Availability

Anonymized patient data will be provided by the corresponding author upon reasonable request, unless the anonymity of the patient is endangered.
